# Correlation between hypoxia-inducible factor-1α C1772T/G1790A polymorphisms and head and neck cancer risk: a meta-analysis

**DOI:** 10.1186/s12957-021-02324-0

**Published:** 2021-07-13

**Authors:** Ting Wu, Zhong-ti Zhang, Lin Li, Ru-yue Liu, Bao-ting Bei

**Affiliations:** VIP Department, School of Stomatology, China Medical University, 117 North Nanjing Street, He ping District, Shenyang, 110002 Liaoning China

**Keywords:** HIF-1α, Head and neck cancer, Polymorphism, Risk, Meta-analysis

## Abstract

**Objective:**

This meta-analysis was implemented to evaluate the association between hypoxia-inducible factor-1α (HIF-1α) C1772T/G1790A polymorphisms and susceptibility to head and neck cancer (HNC).

**Material and methods:**

This meta-analysis has been registered on PROSPERO platform (CRD42021257309). The PubMed, Embase and Web of Science databases were searched to retrieve eligible published papers. STATA software was used to calculate the pooled odds ratios (ORs) and corresponding 95% confidence intervals (CIs) to assess the correlation strength.

**Results:**

Our results demonstrated that the HIF-1α C1772T polymorphism was significantly related to an increased HNC risk (OR = 2.27, 95% CI = 1.17–4.42 for the homozygous model; OR = 11.53, 95% CI = 1.11–120.4 for the recessive model), especially in Caucasians (OR = 2.16, 95% CI = 1.09–4.27 for the homozygous model; OR = 2.28, 95% CI = 1.15–5.51 for the recessive model). Similarly, a remarkable correlation was discovered between the G1790A polymorphism and HNC risk (OR = 72.11, 95% CI = 2.08–2502.4 for the homozygous model; OR = 58.05, 95% CI = 1.70–1985.77 for the recessive model). Moreover, in the subgroup analysis by source of controls, a statistically significant correlation was discovered in the population-based (PB) subgroup (OR = 9.43, 95% CI = 1.20–73.9 for allelic model; OR = 72.11, 95% CI = 2.08–2502.4 for the homozygous model; OR = 3.22, 95% CI = 1.28–8.08 for the heterozygous model; OR = 7.83, 95% CI = 1.48–41.37 for the dominant model; OR = 58.05, 95% CI = 1.70–1985.8 for the recessive model) but not in the hospital-based (HB) subgroup.

**Conclusion:**

Our study found that both HIF-1α C1772T and G1790A polymorphisms might be a higher risk of HNC, especially in the Caucasian group with the C1772T polymorphism.

**Supplementary Information:**

The online version contains supplementary material available at 10.1186/s12957-021-02324-0.

## Introduction

Head and neck cancer (HNC), which includes oropharyngeal cancer, nasopharyngeal cancer, laryngeal cancer and tongue cancer, is the eighth most common cancer according to the latest data reported in the Global Cancer Statistics 2018 [1, 2]. Based on statistics, more than 550,000 new cases of HNC occur worldwide each year, with 300,000 deaths [3]. Although the treatment of HNC patients is progressing, the age of patients has gradually decreased in recent years [4], which may be due to the infection of human papillomavirus (HPV), environmental pollution and unhealthy living habits [5, 6]. Because tumours have low survival rates and high mortality, the quality of life of patients with cancer is greatly reduced [7]. HNC is caused by multiple factors, of which smoking and drinking alcohol are recognized as major risk factors [8–12]. For instance, the study has shown that tobacco increases mutations in cancer [13]. In addition, infection of HPV can cause a variety of cancers, such as cervical cancer and oropharyngeal cancer, and has recently attracted the attention of scientists as another important risk factor for HNC [14, 15]. However, not every individual exposed to the above conditions will have HNC, which indicates that individual genetic susceptibility is also an important factor in the occurrence of HNC [6, 16, 17].

Hypoxia initiates a series of cellular responses, such as angiogenesis, proliferation and glucose and energy metabolism, which might result in the occurrence and development of tumours [18]. Hypoxia-inducible factor-1 (HIF-1) can regulate cellular adaptations to hypoxia [19]. Moreover, it has been reported that HIF-1 can activate numerous genes that play a key role in the critical biological behaviour of tumours [20, 21]. HIF-1α has the ability to determine the activity of HIF-1 and can regulate the expression level of genes related to angiogenesis and metastasis. Many researchers have demonstrated that high HIF-1α expression is found in most human tumours, such as breast carcinoma, hepatocellular cancer, cervical cancer and colorectal tumours [22–25], and HIF-1α may also be a prognostic marker in patients with oral cancer [26]. Moreover, carbonic anhydrase IX, a hypoxia-induced enzyme, is related to HIF-1α activity, as its overexpression is associated with poor prognosis in a variety of tumours, especially neuroblastoma [27]. Under normal oxygenation conditions, HIF-1α is modified by the enzyme prolyl hydroxylase (PHD), bound by von Hippel-Lindau factor (VHL), ubiquitinated and degraded by the proteasome. Alterations of this system predispose patients to a higher susceptibility to the development of tumours caused by mutations inactivating VHL, with a false signal of hypoxia [28]. In addition, recent studies have discovered that HIF-1α is related to poor prognosis in most tumours [29–31].

HIF-1 gene polymorphisms mediate genetic predisposition to cancer, of which C1772T and G1790A are two common single nucleotide polymorphisms (SNPs) of the HIF-1 gene [32]. Both polymorphisms have been reported to result in increased HIF-1α transcription activity under hypoxic conditions [33, 34]. Additionally, it has been reported that both are related to increased cancer microvessel density, and they are crucial in the progression of different cancers [23, 24, 34, 35].

In recent years, several researchers have reported the potential relationship between HIF-1α polymorphisms and susceptibility to HNC, but the results have been conflicting [32, 34, 36–40]. Thus, it is essential to conduct a comprehensive meta-analysis with high statistical power to study the role of HIF-1α C1772T and G1790A polymorphisms in the progression of HNC.

## Methods

### Selection of relevant studies

The meta-analysis was guided in strict accordance with the recommendations of the Preferred Reporting Items for Systematic Reviews and Meta-Analyses statement [41] (Additional file 1). The meta-analysis has been registered on PROSPERO platform (https://www.crd.york.ac.uk/prospero/display_record.php?ID=CRD42021257309) with the registration number CRD42021257309. We used the "PICOs" strategy to guide the development of the research question (P: HNC patients; I: T(C1772T) and A(G1790A); C: C(C1772T) and G(G1790A); O: the risk of HNC; S: case–control study). The computerized literature retrieval was conducted using the PubMed, Embase and Web of Science databases to identify qualified studies with the following terms: ‘hif-1α’, or ‘hypoxia-inducible factor-1α’, or ‘hif-1’, or ‘hypoxia-inducible factor-1’, or ‘rs11549465’, or ‘C1772T’, or ‘P582S’, or ‘rs11549467’, or ‘G1790A’, or ‘A588T’ And ‘mutation’, or ‘mutations’, or ‘variants’, or ‘variant’, or ‘polymorphism’, or ‘polymorphisms’ And ‘carcinoma’, or ‘neoplasm’, or ‘tumour’, or ‘cancer’, or ‘carcinogenesis’ And ‘head and neck’, or ‘HNC’, or ‘oral’, or ‘oral cavity’, or ‘pharyngeal’, or ‘laryngeal’, or ‘laryngopharyngeal’, or ‘hypopharyngeal’, or ‘nasopharyngeal’, or ‘oropharyngeal’. The retrieval time was from database establishment to 5 November, 2020. Finally, all the included studies were carefully reviewed by the researchers (WT and BBT) to determine eligible studies, and another researcher (LL) discussed any differences.

### Inclusion and exclusion criteria

Literature that satisfied the following criteria was included: (1) case-controlled, (2) described the correlation between the polymorphisms of HIF-1α C1772T and G1790A and HNC risk, and (3) data provided by the study were available. Literature that conformed to the following criteria were not enrolled: (1) nonhuman studies, reviews, editorials, commentaries and case reports; (2) full text could not be found; and (3) no sufficient information was provided.

### Data extraction and quality assessment

The following data were retrieved: first author, publication year, country, ethnicity, genotyping methods, sources of controls, counts of case group and control group, genotype and allele frequencies for cases and controls, cancer site and *P* value of Hardy-Weinberg equilibrium (HWE) in controls. The Newcastle-Ottawa Scale (NOS) was selected for literature quality assessment, including population selection, comparability between groups, and exposure factors [42]. The Office of Health Assessment and Translation (OHAT) risk of bias rating tool was applied to evaluate the bias risk of the included articles [43–45]. Data extraction and quality assessment were conducted independently by two researchers (WT and BBT), and disagreements were discussed with a third researcher (LL).

### Statistical analysis

STATA 11.0 (College Station, TX 77845, USA) was used for our meta-analysis. The strength of the correlation between HNC risk and HIF-1α C1772T/G1790A polymorphisms was assessed by ORs along with the corresponding 95% CIs. In our meta-analysis, we examined the relationship using allelic, homozygous, heterozygous, dominant and recessive genetic models. The genotyping method, ethnicity, source of control group and tumour site were used to perform subgroup analysis to determine whether certain factors were correlated with the overall ORs. Cochran's *Q* test and *I*^2^ test were used for heterogeneity analysis. When *P* < 0.10 or *I*^2^ > 50%, we believed that there was heterogeneity among the studies, and the DerSimonian and Laird random effects model was used for analysis; otherwise, the Mantel-Haenszel fixed effect model was used for analysis. Publication bias was evaluated by Begg's test and Egger's test, and the stability of the results was evaluated by sensitivity analysis. A *Z* test was conducted to analyse the statistically significant results, and a *P* value less than 0.05 was regarded as statistically significant.

## Results

### Literature search and characteristics of studies

**Fig. 1:**
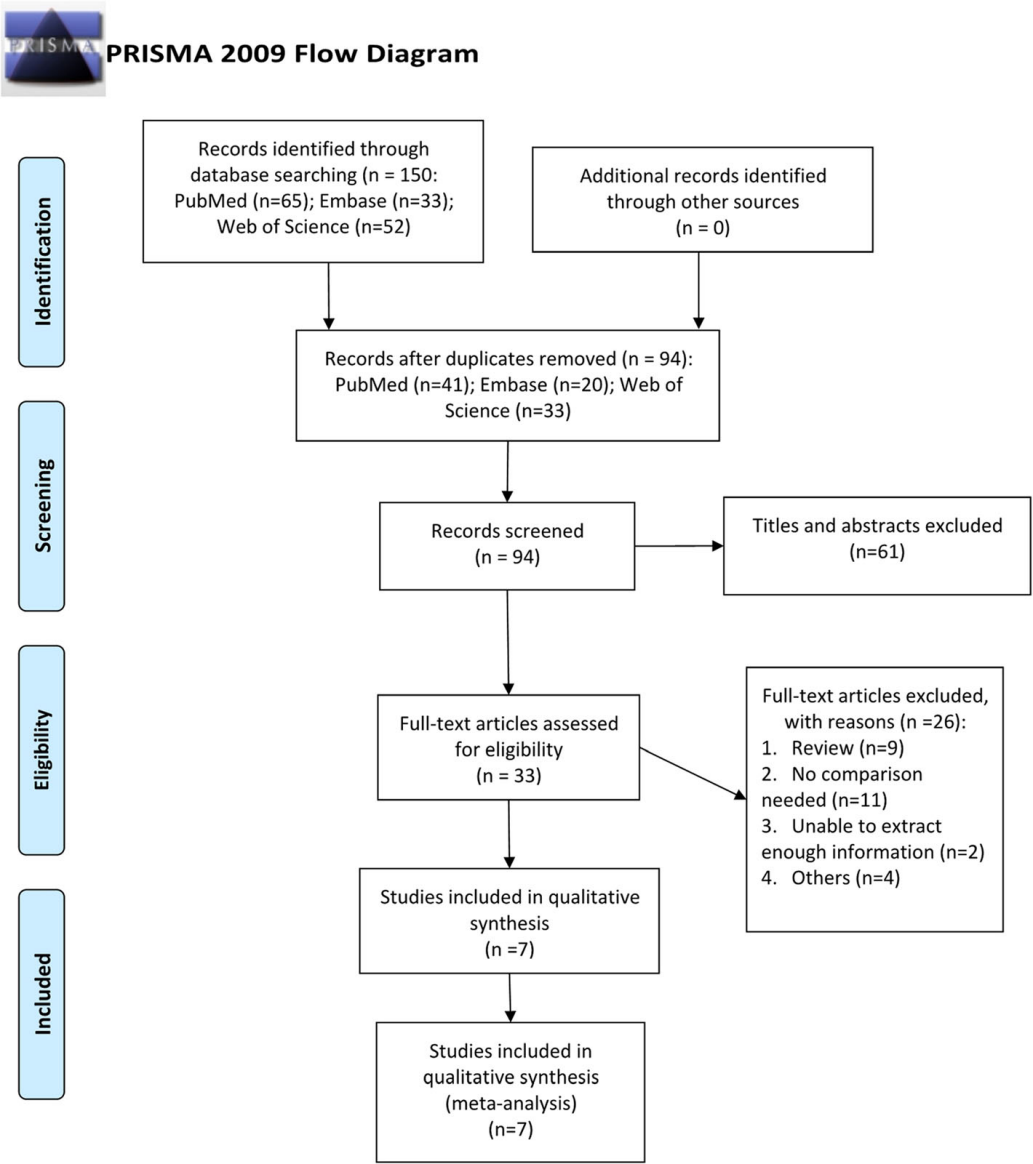
The process of study selection

After excluding animal studies, reviews, repeated studies, conference studies and reading the full text, 7 original studies [32, 34, 36–40] were ultimately included. The article selection process is shown in Fig. 1.

Among the 7 enrolled studies, 7 studies were ultimately correlated with the C1772T polymorphism [32, 34, 36–40], and 6 studies were related to the G1790A polymorphism [30, 32, 37–40]. Overall, the meta-analysis included five articles conducted on oral cancer (OC), one article on glottic laryngeal cancer (GLC), and one on HNC. Of the 7 studies on the C1772T polymorphism, the genotype distributions in the controls in 4 articles complied with HWE, and 3 studies did not [32, 38, 40]. All studies related to the HIF-1α G1790A polymorphism showed that the genotype distribution of the control was in line with HWE. The main characteristics of the enrolled studies are shown in Table 1. The results of the researchers' scoring of the included studies according to the NOS scale are shown in Additional file 2, Table S1. The results of risk of bias assessment according to the OHAT risk of bias rating tool are shown in Additional file 2, Table S2.

**Table 1:** Detailed information of the included articles

First author	Year	Country	Ethnicity	Genotyping method	SC	Case–control	Cases			Controls			Cancer type	HWE
C1772T							CC	CT	TT	CC	CT	TT		
Prasad J	2018	India	Asian	Sequencing	HB	50/50	43	7	0	42	8	0	OSCC	0.539
Alves LR	2012	Brazil	Brazilian	PCR-RFLP	PB	40/88	0	1	39	0	85	3	OSCC	< 0.001
Mera-Menendez F	2012	Spain	Caucasian	PCR-RFLP	HB	118/148	85	18	15	113	27	8	Glottic laryngeal cancer	0.001
Shieh TM	2010	China	Asian	Sequencing	HB	305/96	282	23	0	89	7	0	OSCC	0.711
Chen MK	2009	China	Asian	PCR-RFLP	PB	174/347	163	10	1	334	13	0	OC	0.722
Munoz-Guerra MF	2009	Spain	Caucasian	PCR-RFLP	PB	70/148	57	6	7	113	27	8	OSCC	0.001
Tanimoto K	2003	Japan	Asian	Sequencing	PB	55/110	45	10	0	98	12	0	HNSCC	0.545
G1790A							AA	AG	GG	AA	AG	GG		
Alves LR	2012	Brazil	Brazilian	PCR-RFLP	PB	40/88	37	1	2	0	7	81	OSCC	0.698
Mera-Menendez F	2012	Spain	Caucasian	PCR-RFLP	HB	111/139	0	4	107	0	9	130	Glottic laryngeal cancer	0.693
Shieh TM	2010	China	Asian	Sequencing	HB	305/96	0	24	281	0	7	89	OSCC	0.711
Chen MK	2009	China	Asian	PCR-RFLP	PB	174/347	1	20	153	0	14	333	OC	0.701
Munoz-Guerra MF	2009	Spain	Caucasian	PCR-RFLP	PB	64/139	3	21	40	0	9	130	OSCC	0.693
Tanimoto K	2003	Japan	Asian	Sequencing	PB	55/110	0	4	51	0	9	101	HNSCC	0.655

*SC*, source of control; *OC*, oral cancer; *NC*, nasopharyngeal carcinoma; *HNC*, head and neck cancer; *HB*, hospital-based study; *PB*, population-based study; *HWE*, Hardy-Weinberg equilibrium; *PCR-RFLP*, polymerase chain reaction–restriction fragment length polymorphism; *PCR*, polymerase chain reaction

### Quantitative synthesis

The results of the meta-analysis, namely, the relationship between HIF-1α C1772T and G1790A polymorphisms and HNC, are shown in Table 2.

**Table 2:** Results of overall and subgroup analyses for C1772T and G1790A polymorphisms

	No.	T versus C			TT versus CC			TC versus CC			TT + TC versus CC			TT versus TC + CC		
		OR	95% CI	*P*^(*Z*)^	OR	(95% CI)	*P*^(*z*)^	OR	(95% CI)	*P*^(*z*)^	OR	(95% CI)	*P*^(*z*)^	OR	(95% CI)	*P*^(*z*)^
C1772T																
Overall	7	1.66	0.92–2.99	0.095	2.27	1.17–4.42	0.016	0.98	0.70–1.38	0.914	1.16	0.85–1.59	0.355	11.53	1.11–120.4	0.041
PCR-RFLP	4	2.44	0.90–6.64	0.081	2.27	1.17–4.42	0.016	0.86	0.55–1.34	0.506	1.14	0.78–1.67	0.503	11.53	1.11–120.4	0.041
Sequencing	3	1.20	0.70–2.03	0.506	–	–	–	1.20	0.69–2.09	0.514	1.20	0.69–2.09	0.514	–	–	–
Caucasian	2	1.26	0.84–1.90	0.270	2.16	1.09–4.27	0.028	0.69	0.40–1.17	0.168	1.02	0.66–1.57	0.926	2.28	1.15–5.51	0.018
Asian	4	1.37	0.88–2.13	0.159	–	–	–	1.30	0.82–2.07	0.269	1.34	0.85–2.12	0.213	–	–	–
HB	3	1.31	0.90–1.90	0.162	–	–	–	0.92	0.57–1.48	0.736	1.13	0.73–1.74	0.582	–	–	–
PB	4	2.87	0.82–10.0	0.099	2.01	0.75–5.41	0.168	1.05	0.64–1.73	0.843	1.20	0.76–1.89	0.442	22.82	0.28–1887.8	0.165
OC	5	1.95	0.70–5.43	0.201	2.01	0.75–5.41	0.168	0.89	0.57–1.40	0.612	1.01	0.66–1.54	0.957	22.82	0.28–1887.8	0.165
G1790A	No.	A versus G			AA versus GG			AG versus GG			AA + AG versus GG			AA versus AG + GG		
		OR	95% CI	*P*^(*Z*)^	OR	(95% CI)	*P*^(*z*)^	OR	(95% CI)	*P*^(*z*)^	OR	(95% CI)	*P*^(*z*)^	OR	(95% CI)	*P*^(*z*)^
Overall	6	4.11	0.84–20.15	0.081	72.11	2.08–2502.4	0.018	1.94	0.83–4.55	0.128	3.57	0.97–13.14	0.055	58.05	1.70–1985.8	0.024
PCR-RFLP	4	8.39	0.98–72.1	0.053	72.11	2.08–2502.4	0.018	2.81	0.91–8.72	0.074	7.00	1.18–41.68	0.032	58.05	1.70–1985.8	0.024
Sequencing	2	1.01	0.50–2.03	0.975	–	–	–	1.01	0.50–2.06	0.975	1.01	0.50–2.06	0.975	–	–	–
Caucasian	2	2.18	0.16–30.19	0.562	–	–	–	2.10	0.16–28.19	0.577	2.24	0.15–34.32	0.563	–	–	–
Asian	3	1.59	0.67–3.78	0.294	–	–	–	1.57	0.69–3.58	0.283	1.59	0.67–3.76	0.290	–	–	–
HB	2	0.86	0.43–1.72	0.667				0.85	0.42–1.73	0.660	0.85	0.42–1.73	0.660	–	–	–
PB	4	9.43	1.20–73.9	0.033	72.11	2.08–2502.4	0.018	3.22	1.28–8.08	0.013	7.83	1.48–41.37	0.015	58.05	1.70–1985.8	0.024
OC	4	9.66	1.31–71.15	0.026	72.11	2.08–2502.4	0.018	3.17	1.26–7.92	0.014	7.92	1.58–39.64	0.012	58.05	1.70–1985.8	0.024

*OC*, oral cancer; *HB*, hospital-based study; *PB*, population-based study; *PCR-RFLP*, polymerase chain reaction–restriction fragment length polymorphism

#### HIF-1α C1772T polymorphism analysis

**Fig. 2:**
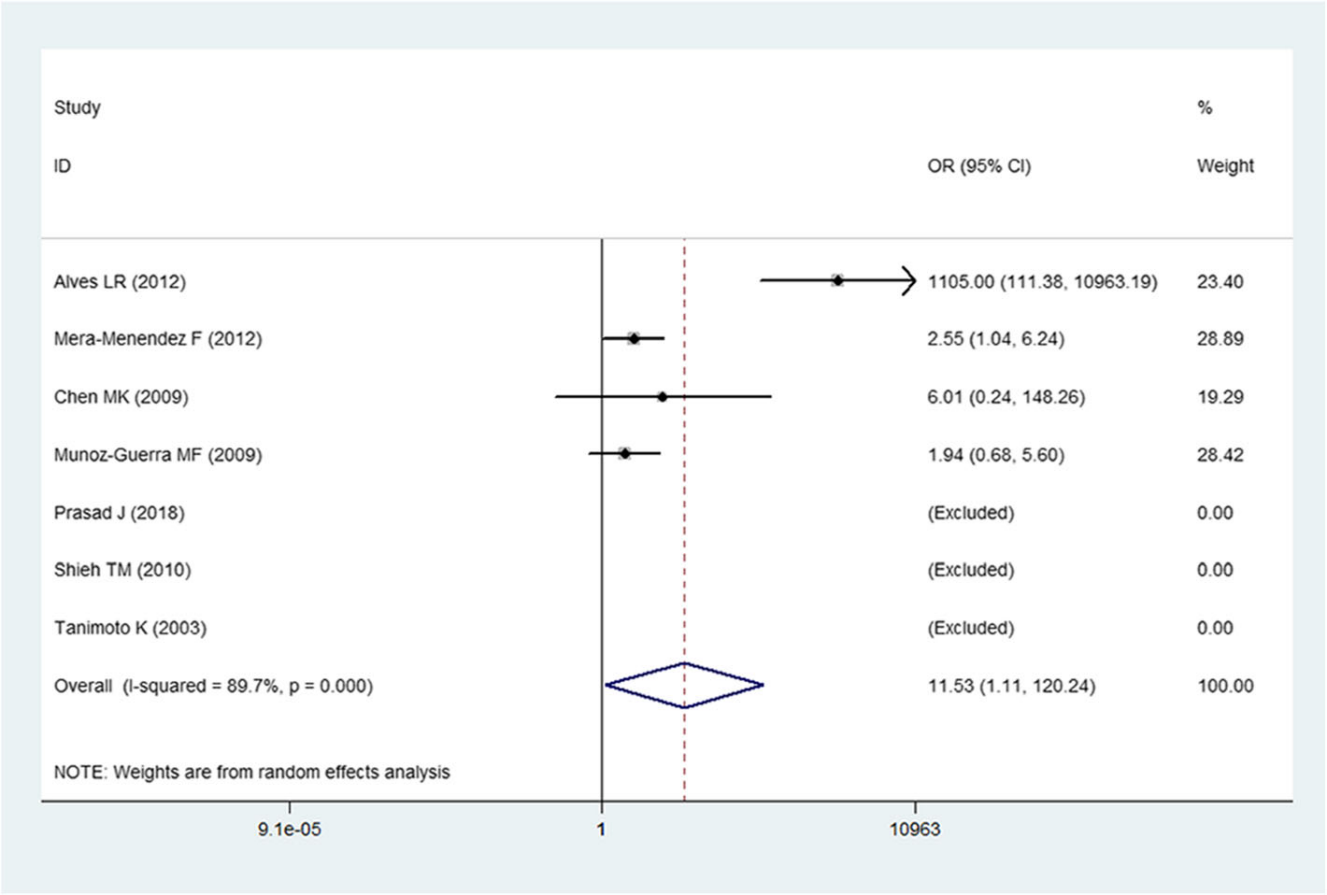
Forest plot for the association of the HIF-1α C1772T polymorphism and HNC risk under a recessive genetic model

For the HIF-1α C1772T polymorphism, a random-effects model was adopted due to the obvious heterogeneity among the studies. We evaluated the association of the C1772T polymorphism with HNC risk in all genetic models except allelic and recessive models. The overall results demonstrated that the C1772T polymorphism was significantly related to a higher HNC risk under the homozygous and recessive genetic models (OR = 2.27, 95% CI = 1.17–4.42 for the homozygous model; OR = 11.53, 95% CI = 1.11–120.4 for the recessive model, Fig. 2) but not under other genetic models (*P* > 0.05). In the subgroup analyses, we found that the C1772T polymorphism could significantly increase HNC risk in the polymerase chain reaction–restriction fragment length polymorphism (PCR-RFLP) genotyping method subgroup (OR = 2.27, 95% CI = 1.17–4.42 for the homozygous model; OR = 11.53, 95% CI = 1.11–120.4 for the recessive model). Moreover, a significant relationship was discovered between the C1772T polymorphism and an increased HNC risk for Caucasians (OR = 2.16, 95% CI = 1.09–4.27 for the homozygous model; OR = 2.28, 95% CI = 1.15–5.51 for the recessive model).

#### HIF-1α G1790A polymorphism analysis

**Fig. 3:**
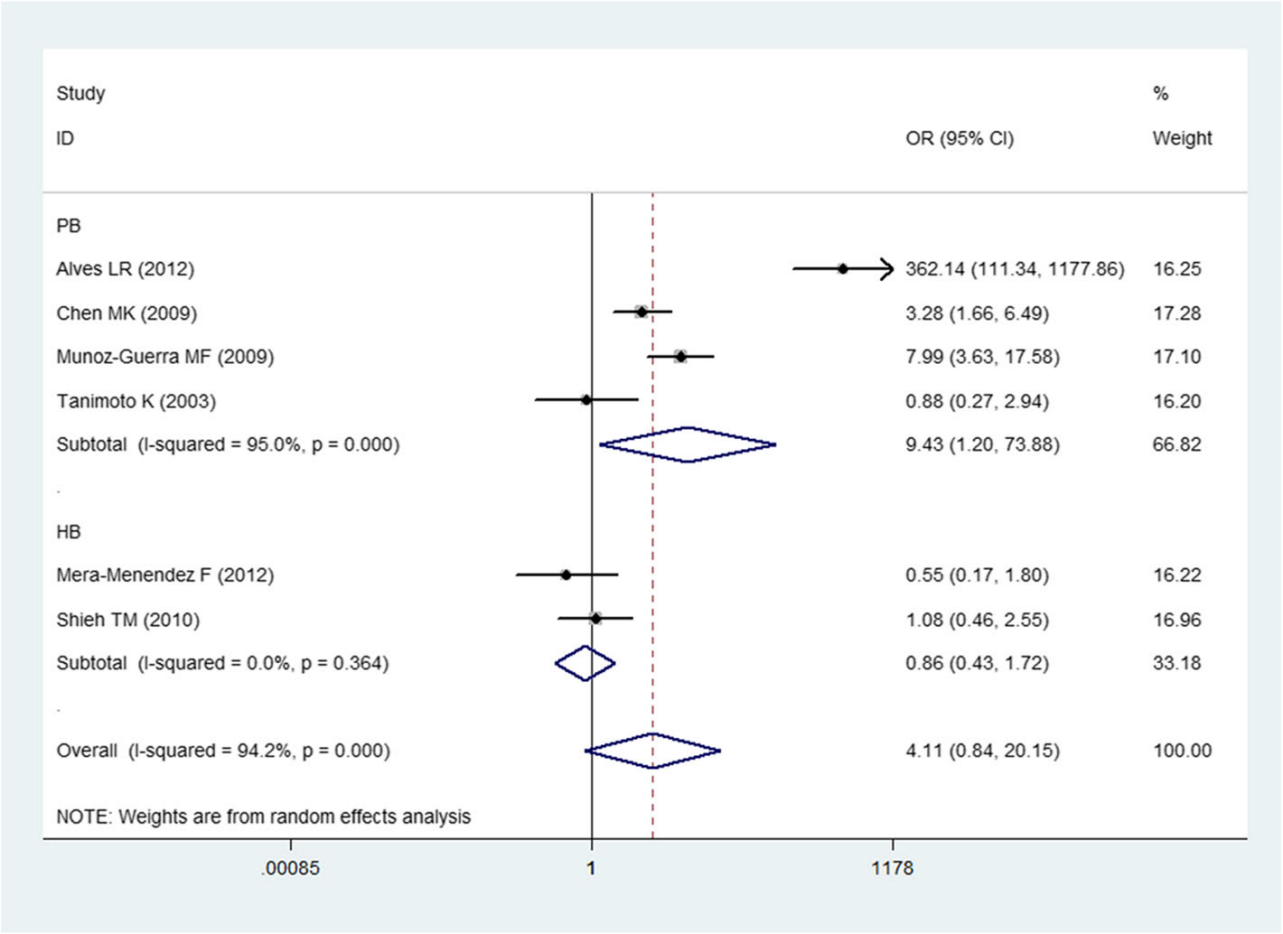
Forest plot for the association of the HIF-1α G1790 polymorphism and HNC risk under the allele genetic model stratified by source of control

For the HIF-1α G1790A polymorphism, we still applied a random effects model to count all the genetic models. We noticed a substantial relationship between the G1790A polymorphism and the increased risk of HNC for the homozygous and recessive genetic models (OR = 72.11, 95% CI = 2.08–2502.4 for the homozygous model; OR = 58.05, 95% CI = 1.70–1985.8 for the recessive model). In the stratified analyses, a substantial relationship was observed for the PCR-RFLP genotyping method subgroup (OR = 72.11, 95% CI = 2.08–2502.4 for the homozygous model; OR = 7.00, 95% CI = 1.18–41.68 for the dominant model; OR = 58.05, 95% CI = 1.70–1985.8 for the recessive model), population-based study subgroup (OR = 9.43, 95% CI = 1.20–73.9 for allelic model, Fig. 3; OR = 72.11, 95% CI = 2.08–2502.4 for the homozygous model; OR = 3.22, 95% CI = 1.28–8.08 for the heterozygous model; OR = 7.83, 95% CI = 1.48–41.37 for the dominant model; OR = 58.05, 95% CI = 1.70–1985.8 for the recessive model) and OC (*P* < 0.05 under all genetic models).

### Sensitivity analysis and publication bias

**Fig. 4:**
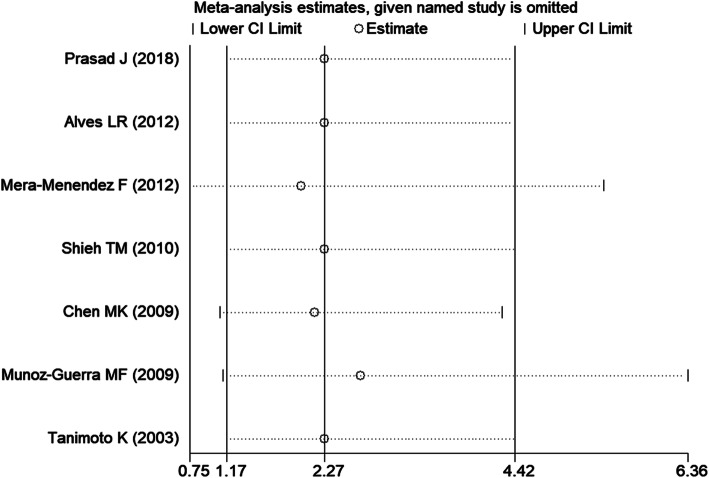
Sensitivity analysis of the pooled OR coefficients on the association of the HIF-1α C1772T polymorphism with HNC risk under a homozygous model

**Fig. 5:**
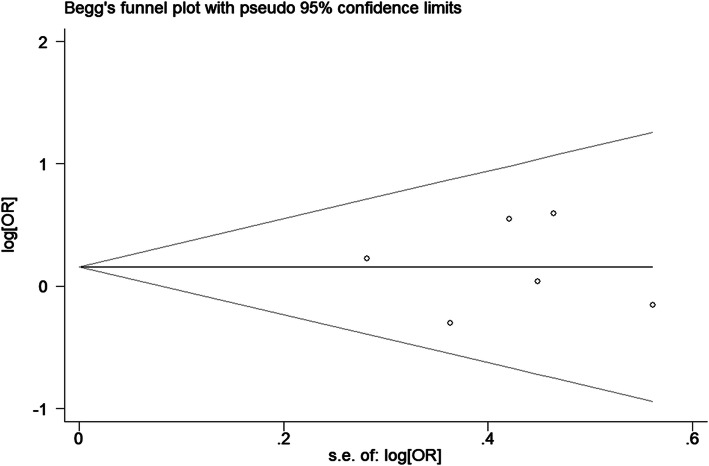
Funnel plot of publication bias for the HIF-1α C1772T polymorphism in HNC under the dominant model

After omitting one article at a time, no significant change was observed in the pooled ORs in the sensitivity analysis (Fig. 4, TT vs. CC of HIF-1α C1772T). Egger tests and Begg’s funnel plots were used to assess publication bias. The *P* value in the Egger test demonstrated statistical evidence for no substantial publication under all genetic models (*P* = 0.188 for T vs. C; *P* = 0.539 for TT vs. CC; *P* = 0.934 for TC vs. CC; *P* = 0.979 for TT + TC vs. CC; *P* = 0.329 for TT vs. TC + CC; *P* = 0.871 for A vs. G; *P* = 0.785 for AA vs. GG; *P* = 0.643 for AG vs. GG; *P* = 0.700 for AA + AG vs. GG; *P* = 0.606 for AA vs. AG + GG). In addition, the shape of Begg’s funnel plot appeared to be symmetric (Fig. 5, TT + TC vs. CC of HIF-1α C1772T), which indicated low risk of publication bias.

## Discussion

HIF-1 acts as a vital component in the progression and metastasis of cancer by activating numerous genes associated with angiogenesis regulation, energy metabolism and cell survival [34, 35]. Moreover, high HIF-1α expression has been demonstrated in various tumours [22–25]. Certain polymorphisms in the HIF-1α gene have been linked to an individual's predisposition to cancers [46]. Among them, HIF-1α C1772T and G1790A polymorphisms were recently confirmed. The potential correlation between HIF-1α C1772T/G1790A polymorphisms and HNC susceptibility has been reported by some investigators, but the results were inconclusive [32, 34, 36–40]. This might be because of the limitations of these studies, such as ethnic differences, control source differences, small sample sizes and different methodologies. Meta-analysis, as a powerful tool, could bridge these difficulties and provide a more precise and reliable conclusion than a single article.

To the best of our knowledge, no studies have evaluated HIF-1αC1772T in the progression of HNC. In this meta-analysis, seven studies were ultimately enrolled for the C1772T polymorphism [32, 34, 36–40], and six studies were included for the G1790A polymorphism [32, 34, 37–40]. Overall, the results demonstrated that the HIF-1α C1772T polymorphism is an important factor in determining the increased risk of HNC (OR = 2.27, 95% CI = 1.17–4.42 for the homozygous model; OR = 11.53, 95% CI = 1.11–120.4 for the recessive model). In addition, a statistically significant correlation between the HIF-1α G1790A polymorphism and a higher risk of HNC was discovered for the homozygous and recessive genetic models (OR = 72.11, 95% CI = 2.08–2502.4 for the homozygous model; OR = 58.05, 95% CI = 1.70–1985.8 for the recessive model). These results indicated that these two polymorphisms play an important role in the progression and development of HNC.

In the stratification analysis of the C1772T polymorphism by the genotyping method, the relevance of the PCR-RFLP genotyping subgroup in the homozygous and recessive models was statistically significant. Regarding the stratification analysis by ethnicity, under homozygous and recessive genetic models, the association between the HIF-1α C1772T polymorphism and increased risk of HNC in the Caucasian population was very significant, but not in the Asian population, which demonstrated genetic diversity among different ethnic groups. This could be explained by the following reasons. First, different ethnic populations live a variety of lifestyles. Second, various environmental factors may be correlated with different ethnicities. Third, various ethnic populations carry different genetic traits.

In the subgroup analysis of the HIF-1α G1790A polymorphism by the genotyping method, we found that there was a statistically significant correlation between PCR-RFLP genotyping method subgroups in the homozygote, dominant and recessive genetic models. This might be because the relationship can be influenced by various genotyping methods, indicating that it is necessary to identify a genotyping method with high specificity and sensitivity to raise the reliability of results. In the subgroup analysis according to the source of controls, a statistically significant correlation was discovered in the PB subgroup but not in the HB subgroup. The reasons for the inconsistent results in HNC risk remain unknown. We supposed that certain selection bias could exist in the HB subgroup because patients without HNC were included, and they might be less representative of the general population than the populations in the PB subgroup. The location of HNC includes the oral cavity, pharynx, cheek and larynx, and different locations have different characteristics; thus, further subgroup analysis was carried out according to tumour types. Regarding the subgroup analysis by tumour type, the HIF-1α G1790A polymorphism was substantially related to a higher risk of OC in the five genetic models. This could be due to different tumour sites being exposed to different microenvironments. HIF-1α expression profiles could be regulated or influenced by the different microenvironments, and the same polymorphism might therefore play different roles in different sites [47].

However, there were some inevitable limitations in this meta-analysis. First, the size of the sample in some subgroups was small, and the results from certain subgroup analyses therefore did not have sufficient power to confirm the relationship. Second, there may have been publication bias because some qualified unpublished articles were not included in our study. Third, subgroup analyses by age, gender, alcohol, smoking or other variables were not performed because of information limitations. Therefore, it is necessary to study the role of HIF-1α C1772T and G1790A polymorphisms in HNC risk with more data and a larger sample size.

Despite these shortcomings, our study has several advantages. First, the latest data were contained in the analysis to evaluate the correlation between C1772T and G1790A polymorphisms in HIF-1α and HNC susceptibility. Second, no publication bias was observed, and evidence for the overall robustness of the results was provided by sensitivity analysis. Additionally, to our knowledge, this is the first meta-analysis to summarize the role of HIF-1α C1772T polymorphisms in HNC susceptibility. However, a previous meta-analysis [48] evaluated the relationship between the G1790A polymorphism and HNC. However, our research has advantages in the following aspects. Our study included five genetic models, and subgroup analyses by genotyping methods and cancer type were performed. Moreover, in this study, we conducted publication bias analysis and sensitivity analysis, which showed that our conclusion was reliable.

## Conclusions

In conclusion, the HIF-1α C1772T and G1790A polymorphisms were significantly related to susceptibility to HNC. Moreover, we found for the first time that the C1772T polymorphism could statistically increase HNC risk among Caucasians. In addition, the HIF-1α G1790A polymorphism was strongly related to a higher risk of HNC, especially OC. However, further well-designed papers with larger sample sizes are needed to confirm our results.

## Supplementary Information

**Additional file 1.** PRISMA 2009 Checklist

**Additional file 2.** Table Supplementary 1. Quality assessment of included studies (Newcastle-Ottawa Scale). Table Supplementary 2. The risk of bias of included studies on the basis of the OHAT.

## Data Availability

The current study was based on the results of relevant published studies.

## Abbreviations

HNC: Head and neck cancer
HIF-1: Hypoxia-inducible factor-1
PHD: Prolyl hydroxylase
VHL: von Hippel-Lindau factor
HWE: Hardy-Weinberg equilibrium
OC: Oral cancer
GLC: Glottic laryngeal cancer
SNPs: Single nucleotide polymorphisms
NOS: Newcastle-Ottawa Scale
OHAT: The Office of Health Assessment and Translation
HPV: Human papillomavirus
