# Supplementary material for: Correlation between hypoxia-inducible factor-1α C1772T/G1790A polymorphisms and head and neck cancer risk: a meta-analysis
Source: World J Surg Oncol. 2021 Jul 13;19:210. doi: 10.1186/s12957-021-02324-0 (PMC8278760; doi:10.1186/s12957-021-02324-0)
Supplement: Supplementary file 1 — Additional file 1. PRISMA 2009 Checklist [file 12957_2021_2324_MOESM1_ESM.pdf]

PRISMA 2009 Checklist

Section/topic	#	Checklist item	Reported on page #
**TITLE**			
Title	1	Correlation between hypoxia-inducible factor-1α polymorphisms and head and neck cancer risk	
**ABSTRACT**			
Structured summary	2	**Abstract**<br>**Objective: We performed a meta-analysis to explore the role of hypoxia-inducible factor-1α (HIF-1α) C1772T/G1790A polymorphisms in the progress of head and neck cancer (HNC).**<br>**Materials and Methods: PubMed, Embase and Web of Science databases were used to retrieve the eligible published papers. Pooled odds ratios (ORs) and corresponding 95% confidence intervals (CIs) were used to evaluate the correlation strength.**<br>**Results: Our results demonstrated that HIF-1α C1772T polymorphism was significantly related to an increased HNC risk (OR=2.27, 95%CI=1.17-4.42 for homozygous model; OR=11.53, 95%CI=1.11-120.4 for recessive model), especially exists in Caucasians (OR=2.16, 95%CI=1.09-4.27 for homozygous model; OR=2.28, 95%CI=1.15-5.51 for recessive model). Similarly, the remarkable correlation was discovered between G1790A polymorphism and HNC risk (OR=72.11, 95%CI=2.08-2502.4 for homozygous model; OR=58.05, 95%CI=1.70-1985.77 for recessive model). Moreover, in the subgroup analysis by source of controls, a statistically significant correlation was discovered in population-based (PB) subgroup, but not in hospital-based (HB) subgroup.**<br>**Conclusion: Our study demonstrated that both HIF-1α C1772T and G1790A polymorphisms might be strongly related to the higher risk of HNC, especially among Caucasion group for C1772T polymorphism.**	
**INTRODUCTION**			
Rationale	3	Our study demonstrated that both HIF-1α C1772T and G1790A polymorphisms might be strongly related to the higher risk of HNC, especially among Caucasion group for C1772T polymorphism.	
Objectives	4	Participants: head and neck cancer<br>Interventions: T(C1772T); A(G1790A)<br>Comparisons: C(C1772T); G(G1790A)<br>Outcomes:	

First Author	Year	Country	Ethnicity	Genotyping Method	SC	Case-Control	Cases			Controls			Cancer Type	HWE
C1772T							CC	CT	TT	CC	CT	TT		
Prasad J	2018	India	Asian	Sequencing	HB	50/50	43	7	0	42	8	0	OSCC	0.539
Alves LR	2012	Brazil	Brazilian	PCR-RFLP	PB	40/88	0	1	39	0	85	3	OSCC	<0.001
Mera-Menendez	2012	Spain	Caucasion	PCR-RFLP	HB	118/148	85	18	15	113	27	8	Glottic laryngeal	0.001

F															cancer	
Shieh TM	2010	China	Asian	Sequencing	HB	305/96	282	23	0	89	7	0	OSCC	0.711
Chen MK	2009	China	Asian	PCR-RFLP	PB	174/347	163	10	1	334	13	0	OC	0.722
Munoz-Guerra MF	2009	Spain	Caucasion	PCR-RFLP	PB	70/148	57	6	7	113	27	8	OSCC	0.001
Tanimoto K	2003	Japan	Asian	Sequencing	PB	55/110	45	10	0	98	12	0	HNSCC	0.545
G1790A							AA	AG	GG	AA	AG	GG		
Alves LR	2012	Brazil	Brazilian	PCR-RFLP	PB	40/88	37	1	2	0	7	81	OSCC	0.698
Mera-Menendez F	2012	Spain	Caucasion	PCR-RFLP	HB	111/139	0	4	107	0	9	130	Glottic laryngeal cancer	0.693
Shieh TM	2010	China	Asian	Sequencing	HB	305/96	0	24	281	0	7	89	OSCC	0.711
Chen MK	2009	China	Asian	PCR-RFLP	PB	174/347	1	20	153	0	14	333	OC	0.701
Munoz-Guerra MF	2009	Spain	Caucasion	PCR-RFLP	PB	64/139	3	21	40	0	9	130	OSCC	0.693
Tanimoto K	2003	Japan	Asian	Sequencing	PB	55/110	0	4	51	0	9	101	HNSCC	0.655

Study design: case-control study.

METHODS

Protocol and registration	5	Indicate if a review protocol exists, if and where it can be accessed (e.g., Web address), and, if available, provide registration information including registration number.	
Eligibility criteria	6	(see table below)	

First Author	Year	Country	Ethnicity	Genotyping Method	SC	Case-Control	Cases			Controls			Cancer Type	HWE
C1772T							CC	CT	TT	CC	CT	TT		
Prasad J	2018	India	Asian	Sequencing	HB	50/50	43	7	0	42	8	0	OSCC	0.539
Alves LR	2012	Brazil	Brazilian	PCR-RFLP	PB	40/88	0	1	39	0	85	3	OSCC	<0.001
Mera-Menendez F	2012	Spain	Caucasion	PCR-RFLP	HB	118/148	85	18	15	113	27	8	Glottic laryngeal cancer	0.001
Shieh TM	2010	China	Asian	Sequencing	HB	305/96	282	23	0	89	7	0	OSCC	0.711
Chen MK	2009	China	Asian	PCR-RFLP	PB	174/347	163	10	1	334	13	0	OC	0.722
Munoz-Guerra MF	2009	Spain	Caucasion	PCR-RFLP	PB	70/148	57	6	7	113	27	8	OSCC	0.001
Tanimoto K	2003	Japan	Asian	Sequencing	PB	55/110	45	10	0	98	12	0	HNSCC	0.545
G1790A							AA	AG	GG	AA	AG	GG		

Alves LR	2012	Brazil	Brazilian	PCR-RFLP	PB	40/88	37	1	2	0	7	81	OSCC	0.698
Mera-Menendez F	2012	Spain	Caucasion	PCR-RFLP	HB	111/139	0	4	107	0	9	130	Glottic laryngeal cancer	0.693
Shieh TM	2010	China	Asian	Sequencing	HB	305/96	0	24	281	0	7	89	OSCC	0.711
Chen MK	2009	China	Asian	PCR-RFLP	PB	174/347	1	20	153	0	14	333	OC	0.701
Munoz-Guerra MF	2009	Spain	Caucasion	PCR-RFLP	PB	64/139	3	21	40	0	9	130	OSCC	0.693
Tanimoto K	2003	Japan	Asian	Sequencing	PB	55/110	0	4	51	0	9	101	HNSCC	0.655

Information sources	7	PubMed, Embase and Web of Science databases were used to retrieve the eligible published papers.	
Search	8	A computerized literature search was conducted by the databases of PubMed, Embase and Web of Science for identifying the qualified studies with the following terms: 'hif-1α' or 'hypoxia-inducible factor-1α' or 'hif-1' or 'hypoxia-inducible factor-1' And 'mutation' or 'mutations' or 'variants' or 'variant' or 'polymorphism' or 'polymorphisms' And 'carcinoma' or 'neoplasm' or 'tumor' or 'cancer' or 'carcinogenesis' And 'head and neck' or 'HNC' or 'oral' or 'oral cavity' or 'pharyngeal' or 'laryngeal' or 'laryngopharyngeal' or 'hypopharyngeal' or 'nasopharyngeal' or 'oropharyngeal'. Finally, we scanned references cited by all the included studies to identify eligible studies.	
Study selection	9	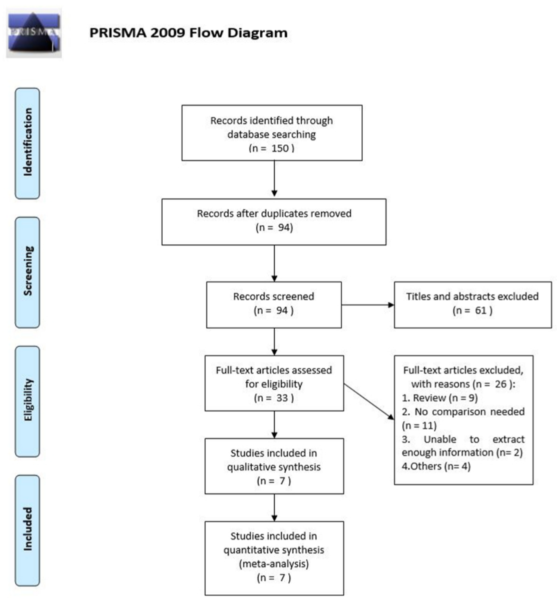	

PRISMA 2009 Checklist

Data collection process	10	Three authors are responsible for extracting data and two authors are responsible for verifying the correctness of the data.	
Data items	11	Data have already been shown in the article.	
Risk of bias in individual studies	12	OHAT risk of bias rating tool was applied to evaluate the bias risk of the included articles.<br>Publication bias analysis and sensitivity analysis have been performed in our study.	
Summary measures	13	State the principal summary measures (e.g., risk ratio, difference in means).	
Synthesis of results	14	Results have already been shown in the article.	

Section/topic	#	Checklist item
Risk of bias across studies	15	No publication bias was found.
Additional analyses	16	Sensitivity and subgroup analyses were performed in our study.
**RESULTS**		
Study selection	17	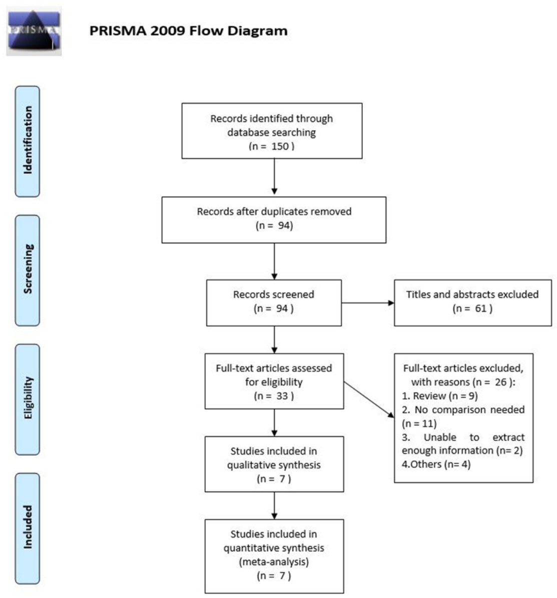

Study characteristics	18	Table 1 The detailed information of included articles

Table 1 The detailed information of included articles

First Author	Year	Country	Ethnicity	Genotyping Method	SC	Case-Control	Cases			Controls			Cancer Type	H
C1772T							CC	CT	TT	CC	CT	TT		
Prasad J	2018	India	Asian	Sequencing	HB	50/50	43	7	0	42	8	0	OSCC	0.
Alves LR	2012	Brazil	Brazilian	PCR-RFLP	PB	40/88	0	1	39	0	85	3	OSCC	<[illegible]
Mera-Menendez F	2012	Spain	Caucasion	PCR-RFLP	HB	118/148	85	18	15	113	27	8	Glottic laryngeal cancer	0.
Shieh TM	2010	China	Asian	Sequencing	HB	305/96	282	23	0	89	7	0	OSCC	0.
Chen MK	2009	China	Asian	PCR-RFLP	PB	174/347	163	10	1	334	13	0	OC	0.
Munoz-Guerra MF	2009	Spain	Caucasion	PCR-RFLP	PB	70/148	57	6	7	113	27	8	OSCC	0.
Tanimoto K	2003	Japan	Asian	Sequencing	PB	55/110	45	10	0	98	12	0	HNSCC	0.
G1790A							AA	AG	GG	AA	AG	GG		
Alves LR	2012	Brazil	Brazilian	PCR-RFLP	PB	40/88	37	1	2	0	7	81	OSCC	0.
Mera-Menendez F	2012	Spain	Caucasion	PCR-RFLP	HB	111/139	0	4	107	0	9	130	Glottic laryngeal cancer	0.
Shieh TM	2010	China	Asian	Sequencing	HB	305/96	0	24	281	0	7	89	OSCC	0.
Chen MK	2009	China	Asian	PCR-RFLP	PB	174/347	1	20	153	0	14	333	OC	0.
Munoz-Guerra MF	2009	Spain	Caucasion	PCR-RFLP	PB	64/139	3	21	40	0	9	130	OSCC	0.
Tanimoto K	2003	Japan	Asian	Sequencing	PB	55/110	0	4	51	0	9	101	HNSCC	0.

FA: First author; SC: source of control; OC: oral cancer; NC: nasopharyngeal carcinoma; HNC: head and neck cancer; HB: hospital-based study; PB: population-basec
HWE: Hardy Weinberg equilibrium; PCR-RFLP: polymerase chain reaction-restriction fragment length polymorphism; PCR: polymerase chain reaction

[28] Munoz-Guerra, M. F., Fernandez-Contreras, M. E., Moreno, A. L., Martin, I. D., Herraez, B., & Gamallo, C. (2009). Polymorphisr hypoxia inducible factor 1-alpha and the impact on the prognosis of early stages of oral cancer. Ann Surg Oncol, 16(8), 2351-23 10.1245/s10434-009-0503-8

[30] Tanimoto, K., Yoshiga, K., Eguchi, H., Kaneyasu, M., Ukon, K., Kumazaki, T., . . . Nishiyama, M. (2003). Hypoxia-inducible facto polymorphisms associated with enhanced transactivation capacity, implying clinical significance. Carcinogenesis, 24(11), 1779-17 10.1093/carcin/bgg132

[32] Prasad, J., Goswami, B., Gowda, S. H., Gupta, N., Kumar, S., Agarwal, K., . . . Chauhan, A. (2018). Does Hypoxia-Inducible Factor (HIF-1alpha) C1772T polymorphism predict short-term prognosis in patients with oral squamous cell carcinoma (OSCC)? J Oral Pat 47(7), 660-664. doi: 10.1111/jop.12718

[33] Shieh, T. M., Chang, K. W., Tu, H. F., Shih, Y. H., Ko, S. Y., Chen, Y. C., & Liu, C. J. (2010). Association between the polymorp exon 12 of hypoxia-inducible factor-1alpha and the clinicopathological features of oral squamous cell carcinoma. Oral Oncol, 46(9), e47 10.1016/j.oraloncology.2010.04.009

[34] Mera-Menendez, F., Hinojar-Gutierrez, A., Guijarro Rojas, M., de Gregorio, J. G., Mera-Menendez, E., Sanchez, J. J., . . . Gamallo, C Polymorphisms in HIF-1alpha affect presence of lymph node metastasis and can influence tumor size in squamous-cell carcinoma of th

larynx. Clin Transl Oncol, 15(5), 358-363. doi: 10.1007/s12094-012-0930-z
[35] Chen, M. K., Chiou, H. L., Su, S. C., Chung, T. T., Tseng, H. C., Tsai, H. T., & Yang, S. F. (2009). The association between inducible factor-1alpha gene polymorphisms and increased susceptibility to oral cancer. Oral Oncol, 45(12), e222-22 10.1016/j.oraloncology.2009.07.015
[36] Alves, L. R., Fraga, C. A. C., Oliveira, M. V. M., Sousa, A. A., Jorge, A. S. B., Marques-Silva, L., Santos, S. H. S., . . . Guimarães, (2012). High HIF-1α expression genotypes increase odds ratio of oral cancer. Head Neck Oncol 4: 2–7

Risk of bias within studies	19	Present data on risk of bias of each study and, if available, any outcome level assessment (see item 12).

Results of individual studies | 20

Table 2 Results of overall and subgroups analyses for C1772T and G1790A polymorphisms

C1772T	No	T versus C			TT versus CC			TC versus CC			TT + TC versus CC			TT versus TC + CC	
		OR	95%CI	$P^{(Z)}$	OR	(95%CI)	$P^{(z)}$	OR	(95%CI)	$P^{(z)}$	OR	(95%CI)	$P^{(z)}$	OR	(95%CI)
Overall	7	1.66	0.92-2.99	0.095	2.27	1.17-4.42	0.016	0.98	0.70-1.38	0.914	1.16	0.85-1.59	0.355	11.53	1.11-120.4
PCR-RFLP	4	2.44	0.90-6.64	0.081	2.27	1.17-4.42	0.016	0.86	0.55-1.34	0.506	1.14	0.78-1.67	0.503	11.53	1.11-120.4
Sequencing	3	1.20	0.70-2.03	0.506	—	—	—	1.20	0.69-2.09	0.514	1.20	0.69-2.09	0.514	—	—
Caucasian	2	1.26	0.84-1.90	0.270	2.16	1.09-4.27	0.028	0.69	0.40-1.17	0.168	1.02	0.66-1.57	0.926	2.28	1.15-5.51
Asian	4	1.37	0.88-2.13	0.159	—	—	—	1.30	0.82-2.07	0.269	1.34	0.85-2.12	0.213	—	—
HB	3	1.31	0.90-1.90	0.162	—	—	—	0.92	0.57-1.48	0.736	1.13	0.73-1.74	0.582	—	—
PB	4	2.87	0.82-10.0	0.099	2.01	0.75-5.41	0.168	1.05	0.64-1.73	0.843	1.20	0.76-1.89	0.442	22.82	0.28-1887.8
OC	5	1.95	0.70-5.43	0.201	2.01	0.75-5.41	0.168	0.89	0.57-1.40	0.612	1.01	0.66-1.54	0.957	22.82	0.28-1887.8
G1790A	No	A versus G			AA versus GG			AG versus GG			AA + AG versus GG			AA versus AG + GG	
		OR	95%CI	$P^{(Z)}$	OR	(95%CI)	$P^{(z)}$	OR	(95%CI)	$P^{(z)}$	OR	(95%CI)	$P^{(z)}$	OR	(95%CI)
Overall	6	4.11	0.84-20.15	0.081	72.11	2.08-2502.4	0.018	1.94	0.83-4.55	0.128	3.57	0.97-13.14	0.055	58.05	1.70-1985.8
PCR-RFLP	4	8.39	0.98-72.1	0.053	72.11	2.08-2502.4	0.018	2.81	0.91-8.72	0.074	7.00	1.18-41.68	0.032	58.05	1.70-1985.8
Sequencing	2	1.01	0.50-2.03	0.975	—	—	—	1.01	0.50-2.06	0.975	1.01	0.50-2.06	0.975	—	—
Caucasian	2	2.18	0.16-30.19	0.562	—	—	—	2.10	0.16-28.19	0.577	2.24	0.15-34.32	0.563	—	—
Asian	3	1.59	0.67-3.78	0.294	—	—	—	1.57	0.69-3.58	0.283	1.59	0.67-3.76	0.290	—	—
HB	2	0.86	0.43-1.72	0.667	—	—	—	0.85	0.42-1.73	0.660	0.85	0.42-1.73	0.660	—	—
PB	4	9.43	1.20-73.9	0.033	72.11	2.08-2502.4	0.018	3.22	1.28-8.08	0.013	7.83	1.48-41.37	0.015	58.05	1.70-1985.8
OC	4	9.66	1.31-71.15	0.026	72.11	2.08-2502.4	0.018	3.17	1.26-7.92	0.014	7.92	1.58-39.64	0.012	58.05	1.70-1985.8

Forest plots were shown in the article.

Synthesis of results | 21

Table 2 Results of overall and subgroups analyses for C1772T and G1790A polymorphisms

C1772T	No	T versus C	TT versus CC	TC versus CC	TT + TC versus CC	TT versus TC + CC

		OR	95%CI	$P^{(Z)}$	OR	(95%CI)	$P^{(z)}$	OR	(95%CI)	$P^{(z)}$	OR	(95%CI)	$P^{(z)}$	OR	(95%CI)
Overall	7	1.66	0.92-2.99	0.095	2.27	1.17-4.42	0.016	0.98	0.70-1.38	0.914	1.16	0.85-1.59	0.355	11.53	1.11-120.4
PCR-RFLP	4	2.44	0.90-6.64	0.081	2.27	1.17-4.42	0.016	0.86	0.55-1.34	0.506	1.14	0.78-1.67	0.503	11.53	1.11-120.4
Sequencing	3	1.20	0.70-2.03	0.506	—	—	—	1.20	0.69-2.09	0.514	1.20	0.69-2.09	0.514	—	—
Caucasian	2	1.26	0.84-1.90	0.270	2.16	1.09-4.27	0.028	0.69	0.40-1.17	0.168	1.02	0.66-1.57	0.926	2.28	1.15-5.51
Asian	4	1.37	0.88-2.13	0.159	—	—	—	1.30	0.82-2.07	0.269	1.34	0.85-2.12	0.213	—	—
HB	3	1.31	0.90-1.90	0.162	—	—	—	0.92	0.57-1.48	0.736	1.13	0.73-1.74	0.582	—	—
PB	4	2.87	0.82-10.0	0.099	2.01	0.75-5.41	0.168	1.05	0.64-1.73	0.843	1.20	0.76-1.89	0.442	22.82	0.28-1887.8
OC	5	1.95	0.70-5.43	0.201	2.01	0.75-5.41	0.168	0.89	0.57-1.40	0.612	1.01	0.66-1.54	0.957	22.82	0.28-1887.8
G1790A	No	A versus G			AA versus GG			AG versus GG			AA + AG versus GG			AA versus AG + GG	
		OR	95%CI	$P^{(Z)}$	OR	(95%CI)	$P^{(z)}$	OR	(95%CI)	$P^{(z)}$	OR	(95%CI)	$P^{(z)}$	OR	(95%CI)
Overall	6	4.11	0.84-20.15	0.081	72.11	2.08-2502.4	0.018	1.94	0.83-4.55	0.128	3.57	0.97-13.14	0.055	58.05	1.70-1985.8
PCR-RFLP	4	8.39	0.98-72.1	0.053	72.11	2.08-2502.4	0.018	2.81	0.91-8.72	0.074	7.00	1.18-41.68	0.032	58.05	1.70-1985.8
Sequencing	2	1.01	0.50-2.03	0.975	—	—	—	1.01	0.50-2.06	0.975	1.01	0.50-2.06	0.975	—	—
Caucasian	2	2.18	0.16-30.19	0.562	—	—	—	2.10	0.16-28.19	0.577	2.24	0.15-34.32	0.563	—	—
Asian	3	1.59	0.67-3.78	0.294	—	—	—	1.57	0.69-3.58	0.283	1.59	0.67-3.76	0.290	—	—
HB	2	0.86	0.43-1.72	0.667	—	—	—	0.85	0.42-1.73	0.660	0.85	0.42-1.73	0.660	—	—
PB	4	9.43	1.20-73.9	0.033	72.11	2.08-2502.4	0.018	3.22	1.28-8.08	0.013	7.83	1.48-41.37	0.015	58.05	1.70-1985.8
OC	4	9.66	1.31-71.15	0.026	72.11	2.08-2502.4	0.018	3.17	1.26-7.92	0.014	7.92	1.58-39.64	0.012	58.05	1.70-1985.8

Risk of bias across studies	22	Present results of any assessment of risk of bias across studies (see Item 15).
Additional analysis	23	In the sensitivity analysis, no remarkable change was observed in the pooled ORs after omitting one article at a time.<br>In the subgroup analyses of C1772T polymorphism, we found C1772T polymorphism could increase the HNC risk significantly in the poly chain reaction-restriction fragment length polymorphism (PCR-RFLP) genotyping method subgroup (OR = 2.27, 95% CI = 1.17-4.42 for homozygous model; OR = 11.53, 95% CI = 1.11-120.4 for recessive model). Moreover, a significant relationship could be discovered betw C1772T polymorphism and an increased HNC risk for Caucasians (OR = 2.16, 95% CI = 1.09-4.27 for homozygous model; OR = 2.28, 95 1.15-5.51 for recessive model).<br>In the stratified analyses of G1790A, a substantial relationship was observed for PCR-RFLP genotyping method subgroup (OR = 72.11, 95 2.08-2502.4 for homozygous model; OR = 7.00, 95% CI = 1.18-41.68 for dominant model; OR = 58.05, 95% CI = 1.70-1985.8 for recessiv model), population-based study subgroup (OR = 9.43, 95% CI = 1.20-73.9 for allelic model, Figure 3; OR = 72.11, 95% CI = 2.08-2502.4 f homozygous model; OR = 3.22, 95% CI = 1.28-8.08 for heterozygous model; OR = 7.83, 95% CI = 1.48-41.37 for dominant model; OR = 95% CI = 1.70-1985.8 for recessive model) and OC (P < 0.05 under all genetic models).

DISCUSSION

Summary of evidence	24	The study discovered that HIF-1α C1772T and G1790A polymorphisms were significantly related to the susceptibility to HNC. Moreover, found that C1772T polymorphism could statistically increase the HNC risk among Caucasions at the first time. In addition, HIF-1α G1790 polymorphism was remarkably related to a higher risk of HNC, especially with OC.
Limitations	25	Some inevitable limitations existed in the meta-analysis. Firstly, the sample size in some subgroup was small, so the results from certain analysis could not have sufficient power to confirm the relationship. Secondly, publication bias might exist because several eligible arti have not published were not enrolled in our study. Thirdly, the subgroup analyses by age, gender, alcohol, smoking, or other variables performed because of information limitation. Therefore, it is necessary to study the role of HIF-1α C1772T and G1790A polymorphisms risk with more data and larger sample size.
Conclusions	26	In conclusion, the study discovered that HIF-1α C1772T and G1790A polymorphisms were significantly related to the susceptibility Moreover, we found that C1772T polymorphism could statistically increase the HNC risk among Caucasions at the first time. In addition G1790A polymorphism was remarkably related to a higher risk of HNC, especially with OC. However, further well-designed papers w sample size are required to confirm our results.
**FUNDING**		
Funding	27	No funding in our paper.

From: Moher D, Liberati A, Tetzlaff J, Altman DG, The PRISMA Group (2009). Preferred Reporting Items for Systematic Reviews and Meta-Analyses: The PRISMA Statement. PLoS Med 6(7): e1000097. doi:10.1371/journal.pmed1000097

For more information, visit: www.prisma-statement.org.
