# Supplementary material for: Correlation between hypoxia-inducible factor-1α C1772T/G1790A polymorphisms and head and neck cancer risk: a meta-analysis
Source: World J Surg Oncol. 2021 Jul 13;19:210. doi: 10.1186/s12957-021-02324-0 (PMC8278760; doi:10.1186/s12957-021-02324-0)
Supplement: Supplementary file 2 — Additional file 2. Table Supplementary 1. Quality assessment of included studies (Newcastle-Ottawa Scale). Table Supplementary 2. The risk of bias of included studies on the basis of the OHAT. [file 12957_2021_2324_MOESM2_ESM.pdf]

Table Supplementary 1. Quality assessment of included studies (Newcastle-Ottawa Scale)

Study	Selection				Comparability	Exposure			Score
	Adequate definition of cases	Representa tiveness of the cases	Selection of control subjects	Definition of control subjects	Control for important factor or additional factor	Exposure assessment	Same method of ascertainm ent for all subjects	Non-respon se rate	
Mera-Menendez F	1	0	0	1	2	1	1	0	6
Shieh TM	1	1	0	1	2	1	1	0	7
Chen MK	1	1	1	1	1	1	1	0	7
Munoz-Guerra MF	1	1	0	1	1	1	1	1	7
Tanimoto K	1	0	1	1	1	1	1	0	6
Prasad J	1	0	0	1	2	1	1	0	6
Alves LR	1	0	0	1	1	1	1	0	5

Table Supplementary 2. The risk of bias of included studies on the basis of the OHAT.

Study	Selection Bias	Confounding Bias	Exclusion Bias	Detection Bias			Other Bias
	Comparison groups	Variable assessment	Outcome data complete	Exposure characterization	Outcome assessment	Outcome reported	
Mera-Menendez F	++	++	+	++	++	++	+
Shieh TM	++	NR	NR	+	+	++	+
Chen MK	++	NR	+	++	++	++	+
Munoz-Guerra MF	++	+	+	++	++	++	+
Tanimoto K	++	NR	NR	++	++	+	+
Prasad J	++	NR	NR	++	++	+	+
Alves LR	++	+	NR	++	++	++	+

++ definitely low risk of bias   + probably low risk of bias   NR probably high risk of bias
